# Effect of Qualitative Feed Restriction in Broiler Breeder Pullets on Stress and Clinical Welfare Indicators

**DOI:** 10.3389/fvets.2020.00316

**Published:** 2020-06-11

**Authors:** Fernanda M. Tahamtani, Hengameh Moradi, Anja B. Riber

**Affiliations:** ^1^Department of Animal Science, Aarhus University, Aarhus, Denmark; ^2^Department of Animal and Poultry Science, College of Aburaihan, University of Tehran, Pakdasht, Iran

**Keywords:** broiler breeder, corticosterone, fault bar, qualitative feed restriction, welfare

## Abstract

The feed restriction applied during rearing of broiler breeders inflicts chronic hunger, and frustration due to unfulfilled behavioural needs for feeding. To alleviate the welfare problems associated with feed restriction, qualitative feed restriction allows a larger amount of feed to be provided without increasing the energy intake. In the present study, the aim was to investigate the effect of scatter-fed qualitative feed restriction on a range of welfare indicators in broiler breeders at the end of the rearing period. In total, 1,200 female breeder chicks of the genotype Ross 308 were housed in 24 pens: six pens of initially 50 birds per dietary treatment. The treatments were: (1) standard feed (Control), (2) standard feed diluted with oat hulls (Insoluble), (3) standard feed diluted with oat hulls and sugar beet pulp (Mixed), and (4) standard feed plus maize silage (Roughage). At 15 weeks of age, a blood sample was taken from 40 birds (10/treatment) five times within 24 h. The plasma was analysed for corticosterone concentration. At 19 weeks of age, a clinical welfare assessment was performed on all birds before they were sacrificed. From each bird, three feathers were plucked and macroscopically examined for the presence of fault bars. Feather length and weight were also recorded. Mortality was registered on occurrence throughout the rearing period. Treatment affected the plumage condition, footpad dermatitis, plumage dirtiness, vent pasting, and number of severe fault bars (*P* ≤ 0.05) but not plasma corticosterone concentration, hock burns, hyperkeratosis and mortality (*P* ≥ 0.17). There was an effect of the interactions between treatment and feather type on the total number of fault bars per feather, average position of the fault bars relative to the base of the feather, and growth rates of feather mass and length (*P* < 0.0001). Overall, the results showed improved welfare of Roughage birds and reduced welfare of Mixed birds, whereas the welfare of Insoluble birds did not seem to differ noticeably from that of Control birds. We recommend to further develop a feeding strategy that includes daily allocation of roughage to broiler breeders during the rearing period.

## Introduction

The welfare of conventional broiler breeders has long been debated, although limited research has addressed this topic. The far majority of research studies on poultry welfare focus on broiler chickens and laying hens. In 2010, the European Food Safety Authority (1) pointed out the top five hazards impacting broiler breeder welfare: inappropriate diet, conventional cages, barren environments, high stocking density and low light intensity (1). The consequences of inappropriate diet are hunger, thirst, outbreaks of feather pecking, and diet-related bone problems. Like their offspring, broiler breeders have a great potential for fast growth (2). Therefore, they are feed restricted throughout life (except for the first week and most severely during the rearing period) to avoid health and reproductive problems arising due to obesity (3, 4). Unfortunately, the feed restriction inflicts chronic hunger and frustration due to unfulfilled behavioural needs for feeding in the broiler breeders (5).

Different strategies have been investigated to alleviate the welfare problems associated with feed restriction and control energy intake. Some of these alternatives include multiple daily meals (6), scatter feeding (7), and qualitative feed restriction (8). A review of the existing knowledge shows that it is clear that the number of daily meals provided, i.e., the applied feeding programme, is likely to affect the behaviour and welfare of the birds (9). However, inconsistency in results from different studies hampers clear recommendations to be derived. Scattering of the feed by use of spin feeders is a practice commonly used during the rearing period of broiler breeders in some parts of the world, particularly Europe and Northern America (1). The scatter-feeding strategy appears to have some, although limited, positive effects on the welfare indicators used to assess hunger (6).

Feed restriction is generally applied by reducing the amount of nutritious feed provided, i.e., quantitative feed restriction. An alternative to this is reducing the energy content in a given amount of feed, i.e., qualitative feed restriction (10). This way, the amount of feed provided can be increased without increasing the total energy intake (8). Consequently, the broiler breeders may reach a higher level of satiety, as the gut content is increased compared to quantitative feed restriction (11, 12). Furthermore, they will spend more time feeding, increasing the likelihood that the behavioural need for feeding may be fulfilled (10, 13–15). When applying qualitative feed restriction, the feed is typically diluted by dietary fibres—either insoluble or soluble fibres [e.g., (16)]. The latter type of fibres can absorb more water thus increasing the intestinal content more than insoluble fibres (17).

Several parameters can be used as indicators of stress and welfare in broiler breeders. For example, feed-restricted broiler breeders may show signs of stress in terms of high levels of plasma corticosterone (5, 18, 19) and high occurrence of fault bars (20). Fault bars are translucent malformations perpendicular to the rachis in the feather that occur during feather growth and are caused by stress experienced by the bird (21). Feather growth itself can also be used as a welfare indicator, as it is affected negatively by insufficient dietary protein levels (22). Furthermore, broiler breeder pullets under qualitative feed restriction (high fibrous diet with an appetitive suppressant) showed good plumage condition, suggesting low feather pecking due to reduced hunger sensation (23). This may also have a positive effect on the skin condition, as an intact plumage protects against skin injuries. However, soluble fibres may increase the risk of wet manure, leaving the litter soiled and moist (16). This increases the risk of contact dermatitis, i.e., footpad lesions and hock burns, and plumage dirtiness (24, 25).

In the present study, the aim was to investigate the effect of feeding programmes including qualitative feed restriction and supplementary roughage on a range of welfare indicators. The control diet (Control) was a commercially available standard feed for broiler breeders. Two of the treatment diets both contained a higher fibre content than the Control diet but differed in one only containing insoluble oat hull fibres (Insoluble) and the other containing a mix of both insoluble oat hulls and soluble sugar beet pulp fibres (Mixed). The last treatment consisted of the control feed supplemented with maize silage as roughage (Roughage). The daily feed allotment among treatments was adjusted based on weekly weighing of the birds for all treatments to reach a similar growth rate. We expected to find a higher plasma corticosterone concentration, more plumage and skin damage, and a higher occurrence of fault bars in the Control diet compared to the other treatments. Furthermore, higher occurrences of contact dermatitis and plumage dirtiness were expected in the treatment Mixed compared to the other treatments. This study was part of a larger study comparing the effects of qualitative feed restriction on a range of other parameters, including undisturbed behaviour in the home pen (Riber et al., submitted), fearfulness and motivation to explore (26), feeding motivation (27), and gut filling and passage time (Steenfeldt et al., in prep).

## Materials and Methods

The experiment was carried out according to the guidelines of the Danish Animal Experiments Inspectorate, Ministry of Environment and Food, Danish Veterinary and Food Administration with respect to animal experimentation and care of animals under study.

### Animals, Housing, and Management

Day-old, non-beak-trimmed, female breeder chicks (*n* = 1,200) of the genotype Ross 308 were acquired via DanHatch A/S from Aviagen, Sweden. The chicks were vaccinated according to the standard procedures of the Danish industry (see Supplementary Material). Upon arrival at the experimental facilities at AU Foulum, Denmark, the birds were individually wing tagged and housed in 24 groups of 50 chicks. The groups were randomly selected, and the weight of the chicks was measured in groups of 12 to ensure that the average weight and the weight variation at the starting point were as equal as possible among all pens. The pens were located in two identical and adjacent rooms with 12 pens in each room. Each pen measured 2 m × 2 m × 2 m (L × W × H) and was covered with wire netting. The initial stocking density was 12.5 birds/m^2^. Five birds per pen were sacrificed for experimental purposes at weeks 5, 10, and 15, resulting in a stocking density of around 10.5 birds/m^2^ from week 5. To keep the stocking density at around 10.5 birds/m^2^, the back wall of each pen was moved 0.25 m into the pen at the end of 10 weeks of age (2 m × 1.75 m) and again at the end of 15 weeks of age (2 m × 1.50 m).

The bottom 70 cm of the sides of each pen was covered by a light-grey sheet of hard plastic to prevent visual contact between individuals from neighbouring pens. The floor of the pens was littered with wood shavings (Røde Softspån, Agroform, same type used in practise). When the litter quality degraded to an unacceptable level in the pens, extra litter was added, or the litter was exchanged. Any interventions with the litter included spreading Stalosan Dry (Vilofoss, Fredericia, Denmark) either on top of the old litter, or, when the litter was exchanged, on the concrete floor before new litter was added. Interventions were done similarly for all pens, even if some pens contained litter of good quality, with the exception that the litter in the Mixed pens was exchanged in week 10 (see description of treatments in the section “Dietary treatments”), whereas the other pens only had extra litter and Stalosan Dry added.

Each pen provided seven water nipples (Ziggity, developed for broiler breeders) which were adjusted in height, as the birds grew, and allowed a water flow of up to 110 ml/min. Water was available 24 h per day for the first 7 days of life and subsequently during the period of light only. Feed was provided by scattering. During the first 3 days, this was mainly done on paper placed underneath the drinking nipples to encourage feeding. The feed was given manually during the first 7 days, and the daily amount allocated was divided in four (days 1+2), three (days 3+4), or two meals (days 5+6+7) per day and scattered on the floor and on paper. During the first 7 days of life, the recommended amounts of feed per bird per day were very close to *ad libitum* intake. From day 8, the birds were fed once a day; a pre-weighed amount of feed was given at 09:00 h from two containers above the pen and thereafter scattered on the floor via four out-lets in the roof of each pen. The containers were filled via an automatic pneumatic system, which allows different feeds and different amounts to be allocated to each pen. The refilling of the container occurred between 9:30 and 10:30 h every day in order to separate in time the sound of the filling from feeding. Scatter feeding was used as this method has been introduced commercially to encourage foraging, prolong feeding and improve uniformity of live weight.

During the first 2 days of life, the light schedule was a 23-h light/1-h dark cycle. On day 2, the light hours were reduced by 1 h/day until a light period of 8 h was reached at 16 days of age. Dawn and dust were included in the dark period and consisted of 20 min each. The light was switched on at 08:00 h and off at 16:00 h. The mean light intensity started at approximately 10 lux. However, the light intensity was reduced to approximately 5–6 lux at 26 days of age due to cannibalistic pecking (see below). The room temperature was set at 33°C at placement and was gradually reduced to 21°C by day 28.

A control pen showed signs of cannibalism at 3 weeks of age, and victims were sprayed with hartshorn oil solution (Pyroleum Animale Crudum, Porcivet from Kruuse, Denmark). In addition, one peck stone (extra hard, 10 kg, Vilofoss) per pen was introduced in all pens at 4 weeks of age. It was placed centrally in the pen and lasted throughout the study. Initially, the anti-pecking treatment eliminated further pecking, but after 2 weeks new incidences of cannibalism occurred. At 7 weeks of age, the birds in the Control pen affected by cannibalism were culled by CO_2_ gassing. A few incidences (*n* = 1–2 per pen) of cannibalism/peck wounds occurred in four other pens. Affected birds were sprayed with the hartshorn oil solution, which brought cannibalism to an end. At the end of the study, the 19 week-old broiler breeder birds were killed by CO_2_ gassing.

### Experimental Treatments

Each of the 24 pens was assigned to one of four treatments such that each treatment had six replicates. This allocation was done in balanced fashion, so that each of the two rooms in which the pens were located had three replicates per treatment. Furthermore, the placement of the pens in each room was done to account for the potential difference in the physical conditions in the rooms (variations in humidity, temperature, activity by the doors vs. in the middle of the barn etc.).

The four dietary treatments used were:

1. Control: standard commercial feed as used in on-farm conditions.
2. Insoluble: standard commercial feed diluted with insoluble fibres (oat hulls).
3. Mixed: standard commercial feed diluted with a combination of insoluble fibres (oat hulls) and soluble fibres (sugar beet pulp).
4. Roughage: standard commercial feed and a provision of roughage (maize silage).

Full diet composition information is provided in Table 1. Throughout the study, the amounts of feed in MJ metabolisable energy (ME) allocated per bird and the feeding programme for the Control birds followed the scheme recommended by DanHatch for broiler breeder pullets. During the experimental period of 19 weeks, the daily amounts of feed (and maize silage) given per treatment were evaluated per week in order to follow the growth curve recommended by Aviagen, though modified by DanHatch. The birds were weighed weekly on a pen basis (in subgroups of 12 birds) until week 18. Daily feed allowance was then adjusted based on the growth of the birds in the previous week to account for reductions in group size due to mortality or birds being removed for testing. Thus, approximately the same amount of daily ME was allocated in all treatments, but due to the differences in fibre content and types the amount of feed allocated differed between treatments. Compared to Control, Insoluble was on average allowed 15.4% larger amounts of feed (min. 5.9%, max. 37.9%), Mixed 9.1% (min. 4.2%, max. 22.1%), and Roughage 14.2% (min. 8.8%, max. 26.2%).

**Table 1:** Diet composition information for the starter 1, starter 2, and grower diets used.

**Diet/Treatment**	**Age**	**Pellet size**	**Metabolisable energy (ME)**	**Protein content**	**Added fibre sources**
		**mm**	**MJ ME/kg**	**g/kg**	
**Starter 1**					
All treatments	Days 1–7	2	11.8	200	N.a.
**Starter 2**					
Control	days 8–42	3.5	10.8	178	N.a.
Roughage	days 8–42	3.5	10.8	178	N.a.
Insoluble	days 8–42	3.5	9.3	152	300 g oh
Mixed	days 8–42	3.5	9.3	153	191 g oh + 25 g/kg sbp
**Grower**					
Control	day 42–week 19	3.5	10.4	145	N.a.
Roughage	day 42–week 19	3.5	10.4	145	N.a.
Insoluble	day 42–week 19	3.5	7.3	110	400 g oh
Mixed	day 42–week 19	3.5	7.5	115	298 g oh + 70 g/kg sbp

*oh, oat hulls; sbp, sugar beet pulp*.

Until day 7, all groups were fed the same standard starter diet 1. A starter diet 2 was provided from day 8 to 42 of age, and thereafter a grower diet was provided. In order to adapt the birds to fibre-rich diets, the starter diet 2 contained less added fibre sources compared with the grower diet (Table 1). For the Roughage treatment, the maize silage was given manually once per day at 9:30 h in two flat, round feeders which were removed again every day at 11:30 h. The amount of maize silage given as a start was 5 g per bird per day.

### Data Collection

Data were collected on a range of welfare indicators, including plasma corticosterone concentration at 15 weeks of age, a clinical welfare assessment at the end of the rearing period (19 weeks of age), occurrence of fault bars on feathers plucked after the birds were culled at 19 weeks of age, and total mortality throughout the rearing period.

#### Plasma Corticosterone Concentration

Plasma corticosterone concentration was measured at 15 weeks of age. For financial reasons, it was only measured once, and 15 weeks of age was chosen as it was within the period in which feed restriction (in terms of amount of feed) is at its most severe level [age 10–16 weeks (10, 28, 29)]. A blood sample was taken from two birds from each treatment at 8:00, 11:00, 16:00, 21:00, and 02:00 h (i.e., two birds per treatment per time point, 40 birds in total). Within a pen, birds were chosen pseudo-randomly by picking the bird nearest to the left side of the peck stone when entering the pen. Immediately after being picked up, a blood sample was taken from the wing vein using a 23-gauge needle (Microlance 3 Kanyle 23Gx 1 ¼, 0.6 mm × 30 mm). The blood samples were centrifuged (1,000 × g for 20 min), marked with bird ID and placed in a freezer (−18°C). The plasma was analysed for corticosterone concentration by a species-independent assay (Catalog Number K014-H, Arbor Assays, 1514 Eisenhower Place, Ann Arbor, Michigan 48108, USA). The instructions given by the manufacturer were followed. Intra- and inter-assay variation were within 8 and 10%, respectively. After blood sampling, the selected birds were sacrificed by the use of CO_2_.

#### Clinical Welfare Assessment

At 19 weeks of age, all birds remaining in the study (*n* = 624; Table 2) were assessed for plumage condition, the presence of skin wounds/scratches, footpad dermatitis, hock burns, hyperkeratosis on the footpads, plumage dirtiness, bumblefoot and vent pasting. The birds were assessed by four experienced observers. Beforehand, the observers were trained together on the protocol used for the present study, including how to differentiate between scores, using live birds and photographs while discussing different cases. Furthermore, the birds in each pen were divided equally between the observers to minimise any potential observer effect. Practically, light was switched on an hour earlier than normal, i.e., at 7:00 h, and birds were fed immediately. At 8:00 h, the light was dimmed in one of the two units, and the two daily caretakers caught the birds in four pens and placed them in crates. The light intensity was then increased to 28 lux, after which the four observers commenced the welfare assessment of their respective pens. When done with one quarter of the birds in a pen, an observer moved to the birds from another of the four pens caught in crates and so forth. Upon completion of the welfare assessment of the birds in the four pens, the birds were humanely killed by the use of CO_2_. The procedure was repeated until all birds in both units had been welfare assessed.

**Table 2:** Number of pens within each treatment of the different group sizes and the mean group size per treatment at 19 weeks of age.

	**Group size**					
**Treatment**	**25**	**26**	**27**	**28**	**29**	**Mean**
Control		2	2	1		26.8
Roughage	1	1	4			26.5
Mixed			3	1	2	27.8
Insoluble			4	2		27.3

The plumage condition was scored using the Bilcik and Keeling (30) method from which the plumage condition of 11 different body parts was scored using a six-point scale. The body parts were head, neck, back, rump, under neck, breast, legs, belly, coverts, tail, and the primary feathers of the wings. The scores ranged from 0 (intact feathers) to 5 (completely denuded area). These 11 body parts as well as the comb and the dorsal side of the feet were inspected for wounds and/or scratches which were noted on a dichotomous scale for each body part (yes/no). Footpad dermatitis was scored on a three-point scale from 0 (no injury) to 2 [serious injury (25)]. Hock burns were scored on a four-point scale from 0 (no injury) to 3 [heavy crust formation on >10% of the hock (31)]. Hyperkeratosis on the footpad [i.e., excessive growth and thickening of the keratin layer of epidermis (32)], bumblefoot [i.e., severe inflammatory state in the subcutaneous tissue causing a bulbous swelling of the footpad (33)] and vent pasting (i.e., excreta adhering to the plumage around the cloaca) were each scored on a dichotomous scale (yes/no). Plumage dirtiness was scored for the ventral part of the body on a four-point scale from 0 (very clean) to 3 (very dirty) according to the Welfare Quality® assessment protocol for poultry (33).

#### Fault Bars

Immediately after killing the birds, three feathers were plucked from each bird: left primary 8 (P8, the third outermost flight feather), rectrix 1 (R1, symmetrical middle tail feather) and left scapular 1 (Sc1, central scapular feather) (20). The three plucked feathers and the wing tag of the bird were placed in a plastic bag and stored in a freezer (−18°C) for later examination. After being thawed, all feathers were macroscopically examined by a single observer for the presence of translucent lines (i.e., fault bars) by holding them against the light. Fault bars were categorised according to the length and severity: (1) minor (<5 mm), (2) moderate (≥5 mm), and (3) severe (≥5 mm and broken barbules on the fault bar) (20). In addition, the position of the fault bar relative to the base of the calamus was measured (digital calliper, ±0.01 mm). Furthermore, the weight of each feather (±0.1 mg) and the total feather length (digital caliper, ±0.01 mm) were recorded. Broken and very dirty feathers were excluded from examination.

#### Mortality

Mortality was registered on occurrence throughout the experimental period, including date, bird ID, pen number, body weight, and the suspected cause of death/reason for culling.

### Statistical Analysis

Statistical analyses were performed using the software SAS 9.3. The concentration of plasma corticosterone was analysed using the mixed procedure and included the fixed factors treatment, the time of day when the blood sample was collected, and the interaction between treatment and time of day. Furthermore, the variable pen was included in the model as a random effect. *Post-hoc* analysis was performed with the Tukey test (Tukey's HSD test).

The plumage scores of each of the 11 body parts were summed for each bird, resulting in a total plumage score ranging from 0 (perfect plumage in all body parts) to 55 (completely denuded in all body parts). The total plumage score was analysed using the mixed procedure with treatment as a fixed effect, and the variables pen and observer as random factors. The individual occurrences of wounds and scratches on each of the body parts assessed were summed per bird and analysed as a whole for each bird. For example, a bird that had wounds on the back, legs, and belly had a final wound score of 3. Nevertheless, as the frequency of these injuries was very low, it was not possible to perform statistical analysis on these data. Therefore, only descriptive statistics are presented.

The data on footpad dermatitis, hock burns and plumage dirtiness were analysed using a multinomial glimmix procedure with treatment as the fixed effect and the variables pen and observer as random factors. The critical *P*-value associated with these analyses was Bonferroni corrected to α = 0.008. The effect of treatment on the incidence of hyperkeratosis was examined using a binary glimmix procedure, also with treatment as the fixed effect, and pen and observer as random factors. The incidence of bumblefoot was very low, and, therefore, only descriptive statistics are presented for this output.

The following variables were analysed from the examination of feathers for fault bars: the total number of fault bars per feather, the total number of severe fault bars per feather, the average bar position relative to the base of the feather, and the growth rate of the feathers both in weight and length per week. The growth rate was estimated by week with the assumption that molting had finished at 12 weeks of age (i.e., growth in weight and length was divided by 7). The analyses of the fault bar data were performed using the mixed procedure and included the fixed effects treatment, feather type (i.e., wing primary, tail, and scapular) and the interaction between treatment and feather type. The models also included bird ID and pen as random effects. Furthermore, the models for growth rate, both in weight and in length, included body weight as a covariate. When needed, raw data were arcsine transformed to meet model assumptions. *Post-hoc* analyses for the main fixed factors were performed with Tukey's test (Tukey HSD test). For the significant interaction effects, the value of the critical alpha was Bonferroni corrected according to the number of interesting comparisons (i.e., α = 0.003 for treatment within feather type comparisons and α = 0.002 for both treatment within feather type and feather type within treatment comparisons).

Mortality for each pen was calculated as a percentage of the flock size at the start (i.e., 50 birds) that died or had to be culled for reasons not related to the data collection. This mortality percentage was compared across treatments with a mixed model with treatment as the fixed factor, and pen was a random factor. The mortality data from the Control pen terminated at 3 weeks of age due to cannibalism were excluded from the analysis.

## Results

### Plasma Corticosterone Concentration

There was no effect of the interaction between treatment and time of the day on the concentration of plasma corticosterone of the birds [mean ± std. dev: 12.24 ± 5.0 ng/ml; *F*_(12,20)_ = 1.16; *P* = 0.37). Neither treatment [*F*_(3,20)_ = 0.17; *P* = 0.92] nor time point [*F*_(4,20)_ = 0.86; *P* = 0.5] affected the plasma corticosterone concentration of broiler breeders.

### Clinical Welfare Assessment

In regard to plumage condition, the body parts with the worst condition were the wings, tail, breast and belly. There was a significant effect of treatment on the total plumage score [*F*_(3,19)_ = 18.12; *P* < 0.0001] with birds from the Mixed treatment having a significantly higher plumage score (i.e., worse plumage condition) compared to the Control birds (*P* = 0.003) and those from the Roughage treatment (*P* < 0.0001; LS mean plumage score ± SE: Control = 9.29 ± 1.8; Insoluble = 11.97 ± 1.8; Mixed = 14.16 ± 1.8; Roughage = 6.15 ± 1.8). Furthermore, the birds from the Roughage treatment had a better plumage condition than those from the Insoluble treatment (*P* = 0.0004) and tended to have a better plumage condition than the Control birds (*P* = 0.074). There was no difference between the birds from the Insoluble treatment and those from the Mixed treatment (*P* = 0.25) or Control birds (*P* = 0.15).

**Figure 1:**
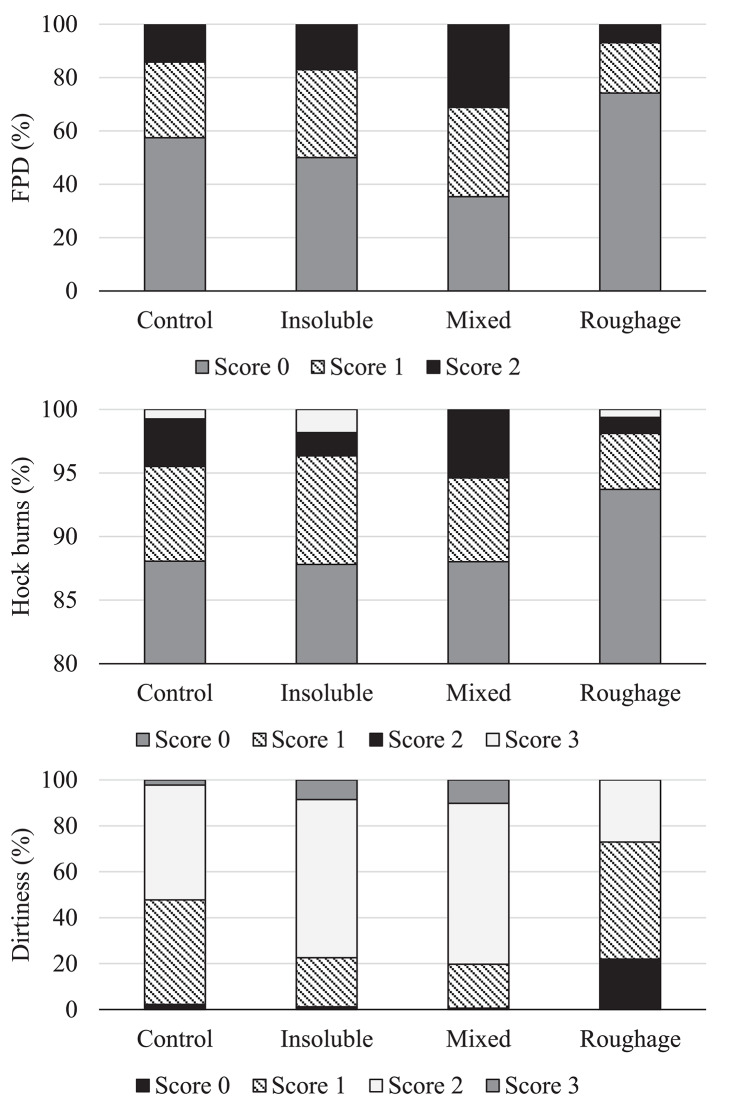
Frequency (%) of footpad dermatitis (FPD), hock burns, and plumage dirtiness scores across treatments. Higher scores represent higher severity of the welfare indicators.

There was an effect of treatment on footpad dermatitis [*F*_(3,597)_ = 4.84; *P* = 0.002] with birds from the Mixed treatment being less likely to have a lower footpad dermatitis score compared to birds from the Roughage treatment (estimated odds = 2.37; *P* = 0.0002; Figure 1). In addition, there was a tendency for birds from the Mixed treatment to have higher footpad dermatitis scores than the Control birds (*P* = 0.04) and for birds from the Insoluble treatment to have higher footpad dermatitis scores than the birds from the Roughage treatment (*P* = 0.03). There was no effect of treatment on hock burn scores [*F*_(3,596)_ = 1.16; *P* = 0.32; Figure 1].

There was an effect of treatment on the plumage dirtiness score [*F*_(3,596)_ = 6.64; *P* = 0.0002; Figure 1] with birds from the Roughage treatment being more likely to have a lower dirtiness score (i.e., cleaner plumage) than birds in the Mixed treatment (estimated odds = 3.99; *P* < 0.0001). Similarly, Roughage birds were more likely to have a lower dirtiness score compared to birds in the Insoluble treatment (estimated odds = 3.72; *P* = 0.0002). There was also a tendency for Roughage birds to have a cleaner plumage than Control birds (*P* = 0.04). The Control birds did, however, not differ from those of the Insoluble or Mixed treatments (*P* > 0.008).

There was an effect of the treatment on the occurrence of vent pasting [*F*_(3,598)_ = 2.62; *P* = 0.05] with the birds from the Roughage treatment tending to be less likely to present with vent pasting compared to the birds from the Insoluble (estimated odds = 2.7; *P* = 0.017) and Mixed treatments (estimated odds = 2.6; *P* = 0.019), respectively. There was no other observed difference between the treatments (prevalence: Control = 3%; Insoluble = 9.8%, Mixed = 9%; Roughage = 0.6%). There was no effect of treatment on the prevalence of hyperkeratosis [*F*_(3,598)_ = 1.42; *P* = 0.23; average prevalence: 28.2%]. The occurrence of wounds and scratches was very low with an average of 0.03 ± 0.17 (mean number of body parts with wounds/scratches ± STD) across treatment groups. The frequency of bumblefoot was very low with only three cases being observed in total: one case in the Insoluble treatment and two in the Mixed treatment.

### Fault Bars

There was an effect of the interaction between treatment and feather type on the total number of fault bars per feather [*F*_(6,1259)_ = 7.45; *P* < 0.0001; Table 3]. Control birds had more fault bars in the tail feathers compared to birds from the Mixed treatment (*P* = 0.0005) but did not differ from the other two treatments. Furthermore, Roughage birds had more fault bars in the tail than birds from the Mixed treatment (*P* = 0.003). In regard to the scapular feathers, Control birds had more fault bars compared to those from the Mixed and Roughage treatments (*P* = 0.003). Finally, there was a tendency for Control birds to have more fault bars in the primary wing feather compared to birds from the Roughage treatment (*P* = 0.01).

**Table 3:** The total number of fault bars per feather for each feather type within treatment (LS means, SE, and back-transformed LS means).

**Feather type**	**Treatment**	**LS means**	**SE**	Back-transformed* LS means
Primary	Control	100.4	3.6	2.8
	Insoluble	88.6	3.2	2.2
	Roughage	81.8	3.4	2.0
	Mixed	84.7	3.2	2.1
Scapular	Control	92.6_b_	4.7	2.4
	Insoluble	74.8_a, b_	3.8	1.7
	Roughage	67.0_a_	4.3	1.5
	Mixed	68.6_a_	3.6	1.5
Tail	Control	108.1_b_	3.6	3.2
	Insoluble	94.4_a, b_	3.3	2.5
	Roughage	105.3_b_	3.3	3.1
	Mixed	85.6_a_	3.5	2.1

a, b *Different letters within feather type indicate significant differences between treatments (P < 0.003)*.

* *Back-transformation: sinh[radians(LS means)] in Microsoft Excel*.

There was an effect of treatment on the number of severe fault bars [*F*_(3,3)_ = 3.49; *P* = 0.02; Table 4] with Control birds having more severe fault bars compared to birds from the Roughage treatment (*P* = 0.02). There was also an effect of feather type [*F*_(2,217)_ = 4.37; *P* = 0.01] with birds having significantly more severe fault bars in the tail feathers compared to the scapular feathers (*P* = 0.01). Furthermore, the scapular feathers also had fewer severe fault bars compared to the primary wing feathers (*P* = 0.04).

**Table 4:** Number of severe fault bars per feather for each treatment (LS means, SE, and back-transformed LS means).

**Parameter**	**LS means**	**SE**	**Back-transformed LS means**
**Treatment**			
Control	95.9_b_	6.2	2.6
Insoluble	78.5_a, b_	7.1	1.8
Roughage	67.1_a_	7.0	1.5
Mixed	75.9_a, b_	5.4	1.8
**Feather type**			
Scapular	67.8_a_	6.2	1.5
Tail	87.7_b_	4.1	2.2
Primary	82.4_b_	2.8	2.0

a, b *Different letters within treatment and within feather type indicate significant differences (P < 0.05)*.

There was an effect of the interaction between treatment and feather type on the average position of the fault bars relative to the base of the feather [*F*_(6,3609)_ = 11.97; *P* < 0.0001] with Control birds and birds from the Roughage treatment having a longer distance between the fault bars and the base of the tail feather than birds from the Mixed treatment (*P* < 0.0001; Table 5).

**Table 5:** Distance (mm) between the fault bars and the base of the feathers for each feather type between treatment (LS means, SE).

**Feather type**	**Treatment**	**LS means (mm)**	**SE**
Primary	Control	78.90	1.40
	Insoluble	81.31	1.21
	Roughage	79.62	1.34
	Mixed	80.77	1.22
Scapular	Control	61.99	2.12
	Insoluble	61.57	1.61
	Roughage	59.80	1.95
	Mixed	60.20	1.50
Tail	Control	61.59_b_	1.33
	Insoluble	57.24_a, b_	1.25
	Roughage	61.85_b_	1.20
	Mixed	52.48_a_	1.34

a, b *Different letters within feather type indicate significant differences between treatments (P < 0.003)*.

There was an effect of the interaction between treatment and feather type on the weekly feather mass growth rate [*F*_(6,3711)_ = 40.36; *P* < 0.0001] with tail feathers from birds in the Mixed treatment having a lower mass growth rate compared to the birds from the other treatments (*P* < 0.002; Table 6). There was also a tendency for the tail feathers of birds from the Roughage treatment to have a higher mass growth rate compared to birds from the Insoluble treatment (*P* = 0.006). Furthermore, across all treatments, primary wing feathers had the highest mass growth rate, scapular feathers had the lowest mass growth rate, and tail feathers had intermediate mass growth rate (*P* < 0.0001).

**Table 6:** Growth rate of the feathers in mass (mg/week) and length (mm/week) for each feather type and treatment (LS means, SE, and back-transformed LS means).

**Feather type**	**Treatment**	**LS means**	**SE**	**Back-transformed LS means**
**Weight**				**(mg/week)**
Primary	Control	246.6	3.4	37.0
	Insoluble	242.7	3.0	34.5
	Roughage	255.6	3.1	43.3
	Mixed	242.1	3.1	34.2
Scapular	Control	147.3	3.6	6.5
	Insoluble	146.5	3.2	6.4
	Roughage	144.7	3.3	6.2
	Mixed	141.1	3.2	5.8
Tail	Control	207.7_b_	3.3	18.8
	Insoluble	192.9_b_	3.0	14.5
	Roughage	209.6_b_	3.0	19.4
	Mixed	174.9_a_	3.1	10.6
**Length**		**(mm/week)**		
Primary	Control	18.9	0.3	N.a.
	Insoluble	19.8	0.3	N.a.
	Roughage	19.0	0.3	N.a.
	Mixed	18.9	0.3	N.a.
Scapular	Control	12.3	0.3	N.a.
	Insoluble	12.5	0.3	N.a.
	Roughage	12.4	0.3	N.a.
	Mixed	12.0	0.3	N.a.
Tail	Control	15.1_b, c_	0.3	N.a.
	Insoluble	13.5_b_	0.3	N.a.
	Roughage	15.4_c_	0.3	N.a.
	Mixed	11.7_a_	0.3	N.a.

a−c *Different letters within growth parameter and feather type indicate significant differences between treatments (P < 0.002)*.

There was an effect of the interaction between treatment and feather type on the feather length growth rate [*F*_(6,3731)_ = 86.97; *P* < 0.0001] with tail feathers from the birds in the Mixed treatment having a lower length growth rate compared to the birds from the other treatments (*P* < 0.0001, Table 6). Furthermore, there was a tendency for the tail feathers of the birds from the Insoluble treatment to have a lower length growth rate compared to Control birds (*P* = 0.005). Across treatments, the length growth rate was highest for the primary wing feathers, lowest for the scapular feathers, and intermediate for the tail feathers, except for the birds from the Mixed treatment. In these birds, the length growth rate did not differ between the scapular and tail feathers (*P* > 0.002).

### Mortality

There was no effect of treatment on mortality [*F*_(3,19)_ = 1.84; *P* = 0.17] with the average mortality during the study period being 4.7%.

## Discussion

The present article reports the results on the effects of four dietary treatments on a range of welfare indicators of female broiler breeders during rearing. The dietary treatments differed in fibre types and content and were all provided by scattering on the litter. These results are part of a larger study, which examined several other parameters such as undisturbed behaviour in the home pen, feeding and exploration motivation, and feed passage rate, all of which are fully presented and discussed elsewhere.

In regard to the effect of qualitative feed restriction on physiological stress parameters in broiler breeders, contradicting results have been found in previous studies on the level of plasma corticosterone (10, 15, 34). Compared to quantitative feed-restricted broiler breeders, Savory et al. (10) only found a reduction in plasma corticosterone concentration when either feeding the birds *ad libitum* standard feed or *ad libitum* feed diluted with 300 g kg^−1^ of oat hulls. In both treatments, the birds grew more than twice as heavy as the target weight within the first 10 weeks of age. At the other extreme, a treatment diet with insoluble fibres in terms of 500 g kg^−1^ of softwood sawdust caused an increase in plasma corticosterone concentration, probably due to a severe suppression of growth rate [about half the target weight at 10 weeks of age (10)]. Furthermore, an increase in plasma corticosterone concentration in a treatment rich in soluble fibres (400 g kg^−1^ of sugar beet) was found, and Savory et al. (10) suggested that the water-holding capacity caused discomfort to the birds. However, as we aimed for a similar growth curve for the different treatments, it was expected that the differences between treatments in the present study would be smaller compared to those found by Savory et al. (10). With a similar growth curve, one can expect that differences in the concentration of corticosterone would be due to differences in stress levels [e.g., due to reduction in frustration in the Insoluble and Mixed treatments vs. the Control (27)] rather than reflect any differences in available energy content of the feed. The overall growth rates of the three treatment diets did, however, all differ slightly from the Control (Riber et al., submitted), but the differences in body weight were only minor at 15 weeks of age when the plasma corticosterone level was measured (Roughage: −0.1%, Insoluble: −2.5%, Mixed: −7.8% compared to Control). However, the pattern of plasma corticosterone concentration might have changed among the treatments with the pullets' age. Furthermore, while there was no treatment effect, there was some variation around the mean, which may suggest that significance could have been found with a larger sample size. Nevertheless, it appears that if the different diets had any effect on satiety and fulfilment of behavioural needs, it was not sufficiently large to affect the level of stress measured as plasma corticosterone concentration. Similarly, van Emous et al. (22) found no effects of feeding a lower content of protein combined with higher fibre content on plasma corticosterone in breeder pullets. The authors explained it as a reflection of a general increase in the content of plasma corticosterone of broiler breeders due to an increased feed restriction during the last 30 years. As a result, the broiler breeders respond less to differences in feed allowance. Indeed, the levels of corticosterone found in the present study were high compared to other studies on broiler breeders (i.e., between 0.5 and 3.5 ng/ml during rearing and under different feeding programmes (8, 28, 35, 36). This may suggest that all treatments induced some stress.

The clinical welfare assessment at 19 weeks of age showed suboptimal conditions of several welfare indicators. This was particularly the case for the birds in the Mixed treatment, whereas the birds in the Roughage treatment had a relatively better welfare. This was evident with regard to plumage condition, plumage dirtiness, footpad dermatitis and vent pasting. Therefore, it seems that the Roughage treatment overcomes some of the negative consequences associated with qualitative feed restriction [e.g., high water intake, poor litter quality, and footpad dermatitis (8, 36)]. The birds from the Insoluble treatment largely did not differ from the Control birds in any of the clinical welfare indicators assessed (i.e., plumage condition, plumage dirtiness, footpad dermatitis, hock burns, vent pasting, or hyperkeratosis).

In the present study, the plumage condition scores were generally low, which prevented analysis of the individual body parts separately. Nevertheless, the treatments did differ in the total plumage condition score. Incomplete feather cover is commonly observed in broiler breeders, which is thought to derive either from feather pecking (23, 36, 37) or from insufficient dietary protein levels affecting feather growth negatively (22). It is well-known from laying hens that availability of appropriate foraging material is a critical factor to prevent the development of feather pecking (38, 39). Furthermore, in broiler breeders, the degree of hunger has been shown to be an important factor for the development of feather pecking (37). This likely explains the improved plumage condition in the birds in the Roughage treatment where maize silage was given daily, providing both a high quality foraging material and a larger amount of feeding material. Nevertheless, the occurrence of feather pecking behaviour in the present study was low and was not observed to differ between the treatments (Riber et al., submitted).

Provision of high quality foraging material stimulates foraging in domestic fowl, including scratching of the litter (40, 41). A high turnover of the litter increases aeration and helps keeping the litter dry by which the litter quality is improved, and the risk of gaining footpad dermatitis and a dirty plumage decreases (42). The negative effects observed from the Mixed treatment are likely to be due to the same mechanisms. The litter in the Mixed treatment had a lower dry content and poorer quality (27), which is a key risk factor for footpad dermatitis (24, 25). Furthermore, a poor litter quality is likely to reduce foraging activities, which was indeed observed, as the Mixed birds were less likely to perform foraging than both Control birds and birds from the Roughage treatment (Riber et al., submitted). Thus, the positive effects of the Roughage treatment on plumage condition and footpad dermatitis indicate that improved fulfilment of the behavioural need for foraging was gained.

As expected, the mortality accumulated over the entire rearing period did not differ between treatments. Interestingly though, of the 17 deaths/culls occurring after the first 9 days of age, cannibalism was the cause of 14 incidences. The majority of the cannibalistic incidences (*n* = 8) occurred in one Control pen in which cannibalism, despite all the applied interventions, could not be deterred from continuation, for which reason we decided to cull the entire pen. The remaining incidences occurred in two Control pens and two Roughage pens, each 1–2 incidences. Studies have previously shown that intensified sensation of hunger may increase the risk of cannibalism, although not consistently (16, 17).

The final welfare indicators investigated were the presence of fault bars and feather growth of different feather types of the birds. Recently, Arrazola and Torrey (20) suggested that these two parameters could be used as welfare indicators in broiler breeders. They showed that exposure to acute, unpredictable stress increased the number of fault bars in primary wing feathers and decreased feather growth in broiler breeder pullets. In the present study, Roughage birds tended to have fewer total fault bars in the wing and scapular feathers and had fewer severe fault bars than control birds. These results indicate that roughage birds may experience less stress during the feather growth period than Control birds, which is in line with the results of the clinical welfare assessment. Furthermore, the birds from the Insoluble treatment did not differ from Control birds in the number of fault bars and tended only to have a lower growth rate of the tail feather length, suggesting limited effect on the stress level experienced by birds in the Insoluble treatment compared to Control birds. In contrast, Arrazola et al. (36) found that broiler breeder pullets fed a diet diluted with 40% soybean hulls (a source of insoluble fibres, dilution rate comparable to the grower diet in the Insoluble treatment in the present study) had fewer fault bars than pullets fed the standard feed. However, it must be noted that feather growth in the present study was calculated with the assumption that molting had finished by 12 weeks of age. Therefore, it is possible that the observed differences in growth rate are in fact due to differences in the time of molting.

Supporting the results from the clinical welfare assessment, a suppressed growth rate of the tail feather length was found for the birds from the Mixed treatment, indicating a negative effect of the Mixed treatment on the welfare of the birds. Only the result from the comparison of the number of fault bars in the birds from the Mixed treatment and the Control birds is more difficult to explain. Birds from the Mixed treatment had fewer fault bars than the Control birds, which in principle was what we had expected if the Mixed treatment proved to have a positive effect on satiety and/or fulfilment of the behavioural need for foraging. However, we found no/limited indications of this in the other welfare indicators investigated (present paper, Riber et al., submitted; (26, 27)), making it difficult to explain the reduced number of fault bars. One possible explanation could be that the reduced growth rate of the feathers in the Mixed treatments resulted in less potential for individual fault bars to be formed separately and, instead, ended up together in single, longer fault bars. Indeed, birds from the Mixed treatment had a shorter mean and a higher variation in the distance between the fault bars and the base of the tail feather compared to the Control birds and the birds from the Roughage treatment. This implies that the stress they experienced occurred not only later in life but also over a more extended period than the stress experienced by the Control birds and the birds from the Roughage treatment. However, while the birds from the Mixed treatment had a numerically higher number of severe fault bars compared to the Control birds, this was not a significant difference. Therefore, it is not possible to affirm that this was the cause of the reduced number of fault bars.

## Conclusion

Overall, the results from both the clinical welfare assessment and the examination of presence of fault bars showed improved welfare in the birds from the Roughage treatment, whereas birds from the Mixed treatment experienced reduced welfare. In contrast, the welfare of the birds from the Insoluble treatment did not seem to differ noticeably from that of the Control birds. Other signs of reduced welfare in the Mixed treatment were also seen in other parameters such as motivation to explore and access litter, interpreted as hunger, and reduced growth [(26, 27) Riber et al., submitted]. There was no difference between the treatments in regards to the plasma corticosterone concentration or the accumulated mortality. A recommendation based on these results would be to further develop a feeding strategy that includes daily allocation of roughage to broiler breeders during the rearing period.

## Data Availability Statement

The datasets generated for this study are available on request to the corresponding author.

## Ethics Statement

The animal study was reviewed and approved by Danish Animal Experiments Inspectorate Danish Veterinary and Food Administration Ministry of Environment and Food.

## Author Contributions

FT participated in the design of the study, carried out data collection and data analysis, and drafted the manuscript. HM carried out data collection, performed data analysis, and drafted the manuscript. AR participated in the design of the study, carried out data collection, and drafted the manuscript. All authors approved the final manuscript.

## Conflict of Interest

The authors declare that the research was conducted in the absence of any commercial or financial relationships that could be construed as a potential conflict of interest.
