# Supplementary material for: Effect of Qualitative Feed Restriction in Broiler Breeder Pullets on Stress and Clinical Welfare Indicators
Source: Front Vet Sci. 2020 Jun 11;7:316. doi: 10.3389/fvets.2020.00316 (PMC7300207; doi:10.3389/fvets.2020.00316)
Supplement: Supplementary file 1 [file Table_1.DOCX]

Supplementary material I: Vaccination programme

| **Age** | **Disease** | **Vaccine** | **Administration route** |
| --- | --- | --- | --- |
| Day-old (hatchery) | Marek’s disease | HVT + Rispens | Injection |
| 1 week (1 or 7 d) | Coccidiosis | Paracox Vet. | Drinking water |
| 2 weeks (11 d) | Infectious Bronchitis, IB | Nobilis IB Ma5 | Spray |
| 3 weeks (25 d) | Newcastle disease, ND | Nobilis ND C2 | Spray |
| 5 weeks (35 d) | Gumboro, IBD | TAD Gumboro vac. Vet | Drinking water |
| 7 weeks (45 d) | Chicken anemia virus infection, CAV | TAD Thymo vac. Vet. | Oral* |
| 8 weeks (56 d) | Infectious Bronchitis, IB | Nobilis IB 4-91 Vet. | Spray |
| 9 weeks (67 d) | Newcastle disease, ND | Nobilis ND C2 | Spray |
| 11 weeks (77 d) | Infectious Bronchitis, IB | Nobilis IB Ma5 Vet. | Spray |
| 13 weeks (91 d) | Avian Encephalomyelitis, AE | A.E. vaccine Nobilis Vet. | Drinking water |
| 16 weeks (105 d) | Avian pneumovirus, TRT | Nobilis Rhino CV | Spray |

** CAV: 1½% of the flock received 10 x doses.*
