# Supplementary material for: Effect of Qualitative Feed Restriction in Broiler Breeder Pullets on Stress and Clinical Welfare Indicators
Source: Front Vet Sci. 2020 Jun 11;7:316. doi: 10.3389/fvets.2020.00316 (PMC7300207; doi:10.3389/fvets.2020.00316)
Supplement: Supplementary file 2 [file Table_2.DOCX]

*Supplementary material II: Average weekly amount of each feed (g) and roughage (g) allocated per treatment.*

| Age (week) | Treatment | Feed (g) | Roughage (g) | Total (g) | Extra amount compared to C (%) |
| --- | --- | --- | --- | --- | --- |
| 1 | Control | 19.6 | 0 | 19.6 | 0 |
| 1 | Insoluble | 19.6 | 0 | 19.6 | 0 |
| 1 | Roughage | 19.6 | 0 | 19.6 | 0 |
| 1 | Mixed | 19.6 | 0 | 19.6 | 0 |
| 2 | Control | 29.0 | 0 | 29.0 | 0 |
| 2 | Insoluble | 29.0 | 0 | 29.0 | 0 |
| 2 | Roughage | 29.0 | 0 | 29.0 | 0 |
| 2 | Mixed | 29.0 | 0 | 29.0 | 0 |
| 3 | Control | 34.6 | 0 | 34.6 | 0 |
| 3 | Insoluble | 34.6 | 0 | 34.6 | 0 |
| 3 | Roughage | 34.6 | 0.9 | 35.4 | 2.5 |
| 3 | Mixed | 34.6 | 0 | 34.6 | 0 |
| 4 | Control | 39.0 | 0 | 39.0 | 0 |
| 4 | Insoluble | 41.3 | 0 | 41.3 | 5.9 |
| 4 | Roughage | 39.0 | 3.4 | 42.4 | 8.8 |
| 4 | Mixed | 41.3 | 0 | 41.3 | 5.9 |
| 5 | Control | 42.7 | 0 | 42.7 | 0 |
| 5 | Insoluble | 49.7 | 0 | 49.7 | 16.4 |
| 5 | Roughage | 42.7 | 4.4 | 47.1 | 10.4 |
| 5 | Mixed | 48.4 | 0 | 48.4 | 13.4 |
| 6 | Control | 46.1 | 0 | 46.1 | 0 |
| 6 | Insoluble | 57.0 | 0 | 57.0 | 23.5 |
| 6 | Roughage | 45.7 | 5 | 50.7 | 9.9 |
| 6 | Mixed | 52.3 | 0 | 52.3 | 13.3 |
| 7 | Control | 49.1 | 0 | 49.1 | 0 |
| 7 | Insoluble | 65.1 | 0 | 65.1 | 32.6 |
| 7 | Roughage | 48.3 | 5.3 | 53.5 | 9.1 |
| 7 | Mixed | 57.9 | 0 | 57.9 | 17.8 |
| 8 | Control | 54.8 | 0 | 54.8 | 0 |
| 8 | Insoluble | 75.5 | 0 | 75.5 | 37.9 |
| 8 | Roughage | 54.2 | 15 | 69.2 | 26.2 |
| 8 | Mixed | 66.9 | 0 | 66.9 | 22.1 |
| 9 | Control | 66.2 | 0 | 66.2 | 0 |
| 9 | Insoluble | 83.5 | 0 | 83.5 | 26.1 |
| 9 | Roughage | 64.6 | 8.9 | 73.4 | 10.9 |
| 9 | Mixed | 76.0 | 0 | 76.0 | 14.8 |
| 10 | Control | 77.6 | 0 | 77.6 | 0 |
| 10 | Insoluble | 88.5 | 0 | 88.5 | 14.0 |
| 10 | Roughage | 78.3 | 10 | 88.3 | 13.8 |
| 10 | Mixed | 83.2 | 0 | 83.2 | 7.1 |
| 11 | Control | 82.2 | 0 | 82.2 | 0 |
| 11 | Insoluble | 90.9 | 0 | 90.9 | 10.7 |
| 11 | Roughage | 82.5 | 12 | 94.5 | 15.0 |
| 11 | Mixed | 86.2 | 0 | 86.2 | 4.9 |
| 12 | Control | 84.3 | 0 | 84.3 | 0 |
| 12 | Insoluble | 92.6 | 0 | 92.6 | 9.9 |
| 12 | Roughage | 84.5 | 12 | 96.5 | 14.5 |
| 12 | Mixed | 87.9 | 0 | 87.9 | 4.3 |
| 13 | Control | 85.3 | 0 | 85.3 | 0 |
| 13 | Insoluble | 93.5 | 0 | 93.5 | 9.6 |
| 13 | Roughage | 85.6 | 12 | 97.6 | 14.4 |
| 13 | Mixed | 88.9 | 0 | 88.9 | 4.2 |
| 14 | Control | 86.2 | 0 | 86.2 | 0 |
| 14 | Insoluble | 94.5 | 0 | 94.5 | 9.6 |
| 14 | Roughage | 86.5 | 12 | 98.5 | 14.3 |
| 14 | Mixed | 89.9 | 0 | 89.9 | 4.3 |
| 15 | Control | 87.4 | 0 | 87.4 | 0 |
| 15 | Insoluble | 95.3 | 0 | 95.3 | 9.1 |
| 15 | Roughage | 87.8 | 12 | 99.8 | 14.2 |
| 15 | Mixed | 91.1 | 0 | 91.1 | 4.3 |
| 16 | Control | 88.4 | 0 | 88.4 | 0 |
| 16 | Insoluble | 96.5 | 0 | 96.5 | 9.1 |
| 16 | Roughage | 88.8 | 15 | 103.8 | 17.5 |
| 16 | Mixed | 92.3 | 0 | 92.3 | 4.4 |
| 17 | Control | 89.1 | 0 | 89.1 | 0 |
| 17 | Insoluble | 97.3 | 0 | 97.3 | 9.3 |
| 17 | Roughage | 89.7 | 15 | 104.7 | 17.5 |
| 17 | Mixed | 93.5 | 0 | 93.5 | 5.0 |
| 18 | Control | 89.3 | 0 | 89.3 | 0 |
| 18 | Insoluble | 98.5 | 0 | 98.5 | 10.2 |
| 18 | Roughage | 89.8 | 15 | 104.8 | 17.3 |
| 18 | Mixed | 94.4 | 0 | 94.4 | 5.6 |
| 19 | Control | 95.4 | 0 | 95.4 | 0 |
| 19 | Insoluble | 108.1 | 0 | 108.1 | 13.4 |
| 19 | Roughage | 93.6 | 15 | 108.6 | 13.9 |
| 19 | Mixed | 108.1 | 0 | 108.1 | 13.4 |
